# A novel dual HDAC and HSP90 inhibitor, MPT0G449, downregulates oncogenic pathways in human acute leukemia in vitro and in vivo

**DOI:** 10.1038/s41389-021-00331-0

**Published:** 2021-05-13

**Authors:** Yi-Wen Wu, Min-Wu Chao, Huang-Ju Tu, Liang-Chieh Chen, Kai-Cheng Hsu, Jing-Ping Liou, Chia-Ron Yang, Shih-Chung Yen, Wei-Chun HuangFu, Shiow-Lin Pan

**Affiliations:** 1 Ph.D. Program for Cancer Molecular Biology and Drug Discovery, College of Medical Science and Technology, Taipei Medical University and Academia Sinica, Taipei, Taiwan; 2 Graduate Institute of Cancer Biology and Drug Discovery, College of Medical Science and Technology, Taipei Medical University, Taipei, Taiwan; 3 School of Pharmacy, College of Medicine, National Taiwan University, Taipei, Taiwan; 4 Warshel Institute for Computational Biology, The Chinese University of Hong Kong, Shenzhen, Guangdong P. R. China; 5 Ph.D. Program for Cancer Molecular Biology and Drug Discovery, College of Medical Science and Technology, Taipei Medical University, Taipei, Taiwan; 6 Ph.D. Program in Drug Discovery and Development Industry, College of Pharmacy, Taipei Medical University, Taipei, Taiwan; 7 TMU Biomedical Commercialization Center, Taipei Medical University, Taipei, Taiwan; 8 TMU Research Center of Cancer Translational Medicine, Taipei Medical University, Taipei, Taiwan; 9 School of Pharmacy, College of Pharmacy, Taipei Medical University, Taipei, Taiwan; 10 School of Life and Health Sciences, The Chinese University of Hong Kong, Shenzhen, Guangdong P. R. China

**Keywords:** Drug discovery and development, Drug development

## Abstract

Acute leukemia is a highly heterogeneous disease; therefore, combination therapy is commonly used for patient treatment. Drug–drug interaction is a major concern of combined therapy; hence, dual/multi-target inhibitors have become a dominant approach for cancer drug development. HDACs and HSP90 are involved in the activation of various oncogenic signaling pathways, including PI3K/AKT/mTOR, JAK/STAT, and RAF/MEK/ERK, which are also highly enriched in acute leukemia gene expression profiles. Therefore, we suggest that dual HDAC and HSP90 inhibitors could represent a novel therapeutic approach for acute leukemia. MPT0G449 is a dual effect inhibitor, and it showed cytotoxic effectiveness in acute leukemia cells. Molecular docking analysis indicated that MPT0G449 possessed dual HDAC and HSP90 inhibitory abilities. Furthermore, MPT0G449 induced G_2_ arrest and caspase-mediated cell apoptosis in acute leukemia cells. The oncogenic signaling molecules AKT, mTOR, STAT3, STAT5, MEK, and ERK were significantly downregulated after MPT0G449 treatment in HL-60 and MOLT-4 cells. In vivo xenograft models confirmed the antitumor activity and showed the upregulation of acetyl-histone H3 and HSP70, biomarkers of pan-HDAC and HSP90 inhibition, with MPT0G449 treatment. These findings suggest that the dual inhibition of HDAC and HSP90 can suppress the expression of oncogenic pathways in acute leukemia, and MPT0G449 represents a novel therapeutic for anticancer treatment.

## Introduction

Acute leukemia is a type of hematologic malignancy caused by the rapid proliferation of abnormal immature blood cells. These leukemia cells often invade organs and the central nervous system due to the rapid accumulation of cancerous cells^1^; therefore, immediate treatment is required for acute leukemia patients. According to the pathological cell type, acute leukemia is referred to as either acute myeloid leukemia (AML) or acute lymphoblastic leukemia (ALL). Over the past 30 years, chemotherapy or a combination of chemotherapeutic agents has played a critical role in cancer treatment; however, the detrimental side effects of this therapy can disrupt the quality of life of patients. Consequently, alternative anticancer therapeutic strategies are urgently needed.

The use of dual or multi-target inhibitors as new therapeutic approaches for anticancer studies has been steadily increasing over the past 5 years. Unpredictable drug–drug interactions, and the dynamic pharmacokinetic and pharmacological issues of combination treatment with two (or more) anticancer agents might lead to unexpected side effects or lower treatment efficacies^2,3^. Therefore, increasing amounts of research have suggested that discovering a drug with dual or multiple molecular targets could improve the balance between therapeutic efficiency and safety, as compared to combined treatments^4,5^.

Histone deacetylases (HDACs) are a family of enzymes that participate in the formation of transcriptional complexes by reversing histone and target protein acetylation to modulate the expression of hematopoiesis-related genes^6^. Currently, there are four pan-HDAC inhibitors, vorinostat, romidepsin, belinostat, and panobinostat, approved by the US Food and Drug Administration (FDA) for leukemia and other hematological malignancies^7–10^. These inhibitors exhibit successful anticancer activity by inducing growth inhibition, cell cycle arrest, and apoptosis^11^. Additionally, pan-HDAC inhibitors have shown synergistic efficacy with other anticancer treatments, such as chemotherapy, radiation therapy, targeted therapy, and immunotherapy, and are widely used in the clinic^11–13^. However, drug–drug interactions and unexpected side effects might diminish the effectiveness of cotreatment. Therefore, we attempted to identify a compound that can target both HDACs and other oncogenic proteins to produce an optimum antitumor effect and decrease unwanted reactions.

Heat shock protein 90 (HSP90) protein is a molecular chaperone that is involved in many cellular processes, including protein folding, molecular transportation, and proteasome-mediated degradation^14^. In cancerous cells, highly expressed oncogenic signaling pathway components, such as PI3K/AKT, JAK/STAT, and oncogenic fusion proteins, have been reported to be cancer-relevant client proteins of HSP90^15–18^. In addition, overexpression of HSP90 has been identified as a poor prognostic marker in lung cancer, esophageal cancer, bladder cancer, melanoma, and leukemia^19–22^. The inhibition of HSP90 allows for client protein destabilization and proteasomal degradation^23–25^. Moreover, preclinical and clinical studies have provided evidence that the combination of HSP90 and pan-HDAC inhibitors exhibits synergistic anticancer effects in various cancer types^26–29^. In order to avoid the problems of combination therapy described above, we developed a dual HDAC and HSP90 inhibitor to investigate anticancer effects in acute leukemia.

In our previous study, we designed a series of dual HDAC/HSP90 inhibitors; in particular, MPT0G449 (compound 26) showed cytotoxicity in lung and colorectal cancer cells and higher selectivity for HDAC and HSP90 enzyme inhibition^30^. In this study, we evaluated the anticancer activity of MPT0G449 in acute leukemia. Based on molecular docking analysis, we revealed that MPT0G449 interacts with HSP90 protein via a 2,4-dihydroxy-5-isopropylbenzoyl moiety structure, and in addition, contains a cap, a linker, and a zinc-binding group structure for HDAC inhibition. We also observed that MPT0G449 induced G_2_ arrest and was involved in cell cycle regulatory protein expression. Moreover, MPT0G449 stimulated mitochondria-mediated cell apoptosis in acute leukemia cells. Furthermore, three major oncogenic pathways, PI3K/AKT/mTOR, JAK/STAT, and MEK/ERK, were downregulated by MPT0G449 treatment. In animal studies, we demonstrated that MPT0G449 strongly suppressed tumor growth and induced the expression of biomarkers (acetyl-histone H3 and HSP70) in xenograft models. Collectively, these results highlight a dual HDAC/HSP90 inhibitory compound, MPT0G449, which represents a new therapeutic strategy for clinical treatment.

## Results

### MPT0G449, a dual HDAC and HSP90 inhibitor, significantly decreases acute leukemia cell viability

We have synthesized a series of dual HDAC and HSP90 inhibitors to examine their anticancer efficacy^30^. Since HDACs and HSP90 are both involved in the critical roles in leukemogenesis, herein, we evaluated the cytotoxic effectiveness of dual HDAC/HSP90 inhibitors. The in vitro cytotoxic effect of the compounds was assessed using MTT assay in acute leukemia cell lines (HL-60 and MOLT-4). As shown in Table 1, compounds MPT0G314, MPT0G448, and MPT0G449 showed higher cytotoxicity than the reference compounds (SAHA and 17-AAG). Notably, MPT0G449 exhibited more remarkable anticancer activity in both leukemic cells, HL-60 (IC_50_ = 0.19 ± 0.04 μM) and MOLT-4 (IC_50_ = 0.11 ± 0.03 μM) than other solid cancer cell lines (IC_50_ = 0.4–1.06 μM)^30^. The comparison of the current series of compounds with the published compounds was shown (Supplementary Table 1).

**Table 1:** The cytotoxic effect of dual HDAC and HSP90 inhibitors in acute leukemia cells.

Compound	Cell lines (IC_50_, mean ± SD, μM)	
	HL60	MOLT-4
**MPT0G313**	>10	2.69 ± 0.34
**MPT0G314**	0.95 ± 0.04	0.75 ± 0.19
**MPT0G315**	1.6 ± 0.13	1.43 ± 0.13
**MPT0G316**	4.88 ± 0.53	2.34 ± 0.34
**MPT0G317**	1.54 ± 0.43	2.72 ± 0.46
**MPT0G446**	4.4 ± 0.14	3.07 ± 0.45
**MPT0G447**	>10	>10
**MPT0G448**	1.02 ± 0.06	0.78 ± 0.25
**MPT0G449**	0.19 ± 0.04	0.11 ± 0.03
**17-AAG**	1.21 ± 0.12	1.42 ± 0.24
**SAHA**	1.32 ± 0.1	1 ± 0.23

In our previous study, MPT0G449 (compound 26) showed high selectivity for HDAC and HSP90α enzyme inhibition (HDAC, IC_50_ = 360.82 ± 17.8 nM; HSP90α, IC_50_ = 77.21 ± 4.27 nM)^30^. These data indicate that MPT0G449 is a dual effect inhibitor that selectively targets pan-HDAC and HSP90, and displays a strong cytotoxic effect in acute leukemia cells. Incidentally, ocular toxicity is one of the side effects of HSP90 inhibition^23,31^. To resolve this, we determined the cell viability of retinal pigmented epithelium cells, ARPE-19, with MPT0G449 treatment. ARPE-19 cells did not show cytotoxic effects after MPT0G449 treatment, and other normal human cells, HUVECs, did not exhibit short-term toxicity under MPT0G449 treatment (Supplementary Table 2).

### Molecular docking analysis of MPT0G499 in HSP90α and HDAC6

**Fig. 1:**
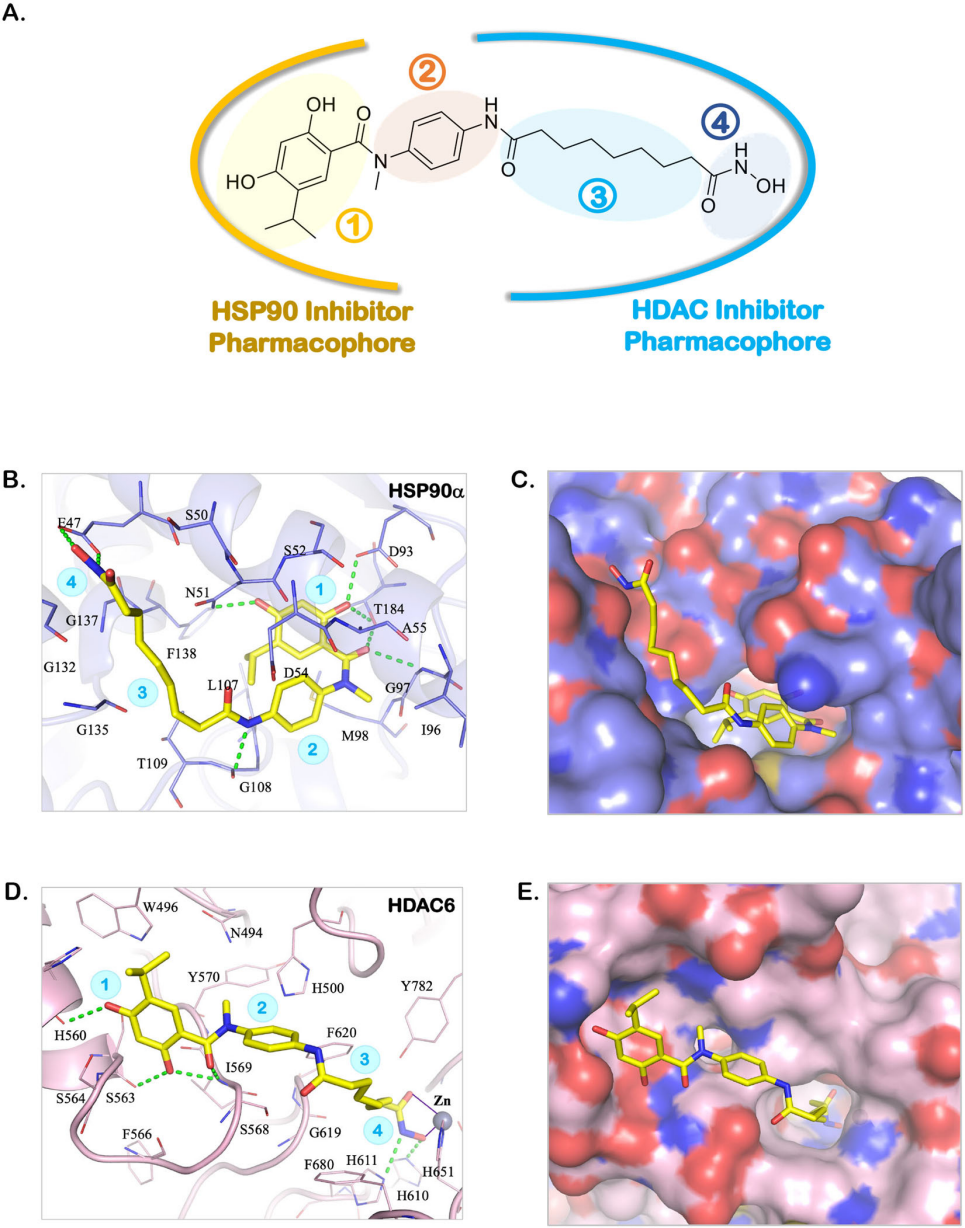
The interaction between MPT0G449 and target proteins analyzes by molecular docking analysis. **A** MPT0G499 can be separated into four groups: 2,4-dihydroxy-5-isopropylbenzoyl (group 1), 4-amino-*N*-methylaniline (group 2), night-carbon chain (group 3) and hydroxamic acid (group 4). Groups 1 is HSP90 inhibitor pharmacophore, while groups 2, 3 and 4 are designed based on the HDAC inhibitor, SAHA. **B** The docking pose of MPT0G499 (yellow) in the HSP90α binding site (purple). Hydrogen bonds are represented as a green dash line. Residues are labeled as shown. **C** The surface model shows that MPT0G499 enters the HSP90α binding site. **D** The docking pose of MPT0G499 (yellow) in the HDAC6 binding site (purple). Hydrogen bonds are represented as a green dash line. Residues are labeled as shown. **E** The surface model shows that MPT0G499 enters the HDAC6 binding site.

MPT0G499 was designed as a dual HDAC and HSP90α inhibitor (Fig. 1A)^30^. The structure of MPT0G499 can be divided into four groups. Group 1 was designed based on an HSP90α inhibitor, AT13387^30,32^, while Groups 2, 3, and 4 were based on SAHA, an FDA-approved pan-HDAC inhibitor (Fig. 1A)^30,33^. To elucidate the interactions of the dual inhibitor, we performed molecular docking analysis against both targets using LeadIT. First, MPT0G499 was docked into the binding site of HSP90α. The docking results showed that Group 1, a 2,4-dihydroxy-5-isopropylbenzoyl moiety, occupies a deep pocket within the binding site. Group 1 forms five hydrogen bonds with residues N51, D93, G97, and T184, and creates hydrophobic interactions with residues N51 and T184 (Fig. 1B). Both Groups 2 and 3 occupied the outer edge of the HSP90α binding site. Group 2 is composed of a 4-amino-*N*-methylaniline moiety and generates a hydrogen bond with residue G108. Group 3 consists of a nine-carbon aliphatic chain that occupies a hydrophobic pocket and interacts with residues M98, T109, and G135 (Fig. 1C). Finally, Group 4 contains a hydroxamic acid moiety that forms two hydrogen bonds with the HSP90α surface residue E47. These interactions suggest that MPT0G499 forms sufficient interactions to inhibit HSP90α activity.

Next, we docked MPT0G499 into the binding site of HDAC6 to analyze its interactions (Fig. 1D). HDAC6 was selected because MPT0G499 exhibited the most potent inhibition. In general, HDAC inhibitors contain three conserved structures: a zinc-binding group (ZBG), a cap group for surface recognition, and a linker that not only connects the above two structures but also spans the hydrophobic tunnel of the binding site^34^. The structure of MPT0G499 conforms to the typical characteristics of HDAC inhibitors, with Groups 1 and 2 functioning as the cap, Group 3 as a linker, and Group 4 as the ZBG. The docking results showed that Group 4 extends deeply into the binding site and coordinates with the zinc ion. It also forms two hydrogen bonds with residues H610 and H611. Group 3 occupies the hydrophobic tunnel, forming a hydrophobic interaction with residue F620. Groups 1 and 2 were located at the periphery of the HDAC6 binding site (Fig. 1E). Group 1 creates four hydrogen bonds with residues H560, S563, and I569, while the aromatic ring of Group 2 generates hydrophobic interactions with residues H500 and S568. These interactions account for the potency of MPT0G499 against HDACs.

### MPT0G449 enhances the expression of the protein acetylation and HSP70 biomarkers in acute leukemia cells

**Fig. 2:**
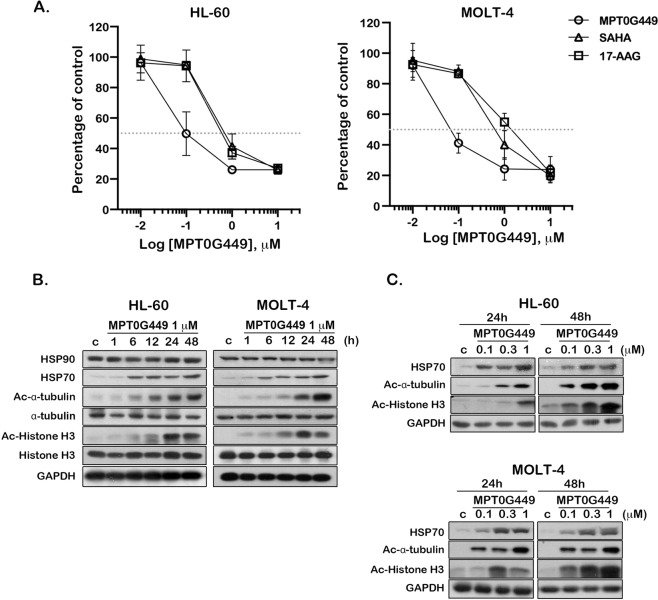
MPT0G449 induces cytotoxicity and inhibits HDAC/HSP90 enzyme activity in acute leukemia cell lines. **A** Cell viability was measured in acute leukemia cell lines, HL-60 and MOLT-4, after 48 h of incubation with MPT0G449, SAHA and 17-AAG (0.01, 0.1, 1, 10 μM), respectively. The results represent the mean ± SD of three independent experiments. **B**, **C** The effect of protein acetylation and HSP70 expression in HL-60 and MOLT-4 cells. Cells were cultured with DMSO (control; c) or indicated concentrations of MPT0G449 for 1, 6, 12, 24, and 48 hours. Whole-cell lysates were detected by western blotting. The data were repeated for at least three independent experiments.

According to the literature, HDACs and HSP90 are overexpressed in various cancer types^35,36^. In multiple leukemia cell lines, we also found that HDAC isoforms and HSP90 proteins were significantly increased compared to normal peripheral blood mononuclear cells (PBMC) (Supplementary Fig. 1A). We then evaluated the impact of the dual effect inhibitor, MPT0G449, on cell viability. The cytotoxic dose–response analysis revealed that both acute leukemia cell lines, HL-60 and MOLT-4, showed higher sensitivity to MPT0G449 when compared to the reference inhibitors (SAHA and 17-AAG) (Fig. 2A). Histone H3, α-tubulin acetylation, and HSP70 are known biomarkers of pan-HDAC and HSP90 inhibition^17,37–39^. MPT0G449 strongly induced acetyl-Histone H3, acetyl-α-tubulin, and HSP70 expression in a time- and concentration-dependent manner (Fig. 2B, C). Furthermore, we demonstrated that MTP0G449 inhibited HDAC enzymes’ activity without altering HDAC proteins (Supplementary Fig. 1B). These results indicate that MPT0G449 exhibits dual HDAC and HSP90 inhibition activities in acute leukemia cells.

### MPT0G449 causes G_2_ arrest in acute leukemia cells

**Fig. 3:**
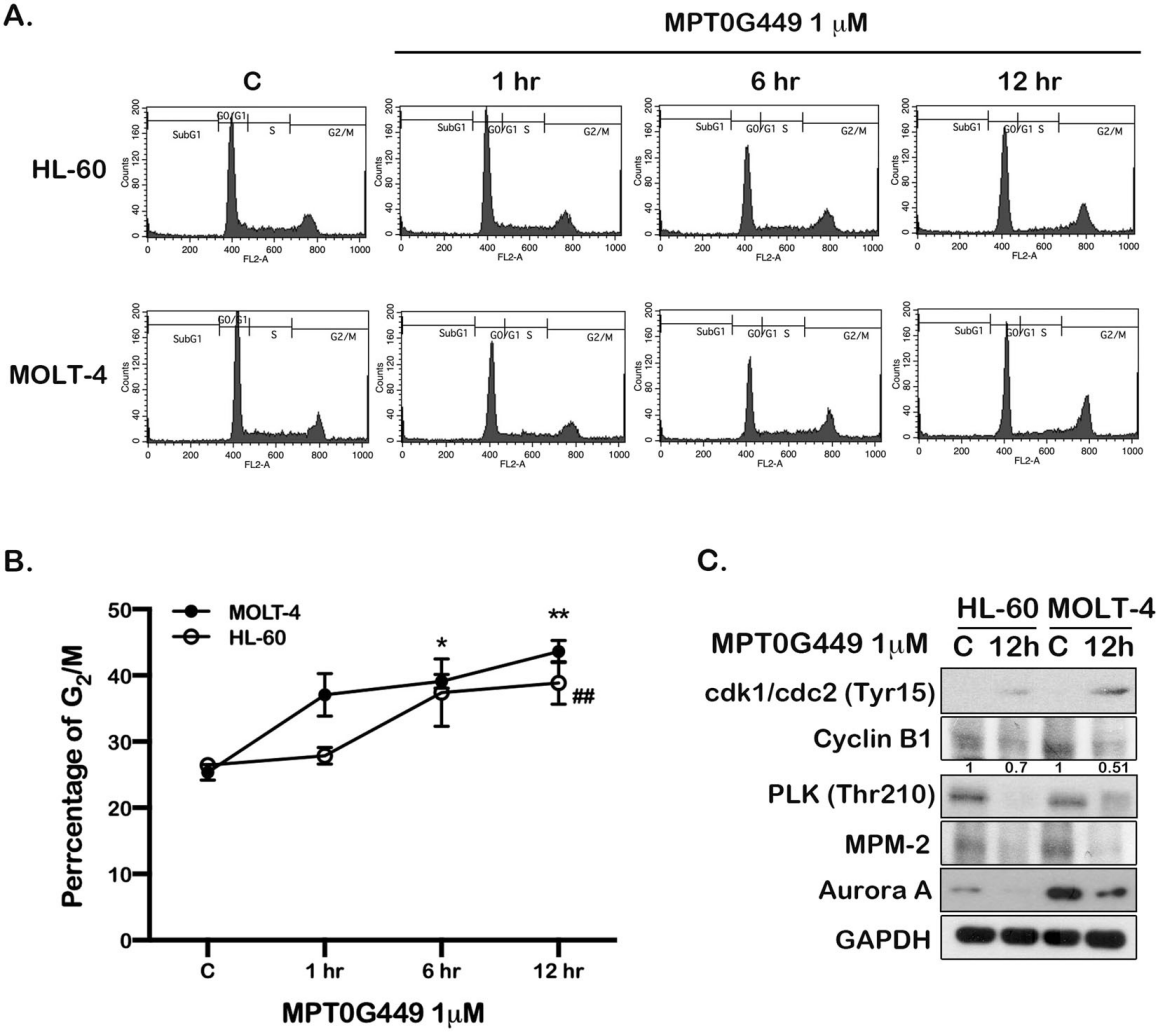
MPT0G449 induces mitotic arrest in HL-60 and MOLT-4. The histograms of cell cycle distribution were detected by flow cytometry. **A** HL-60 and MOLT-4 cells were treated with MPT0G449 (1 μM) for 1, 6, and 12 h. **B** The statistical analysis of G_2_/M profiles. The results represent the mean ± SD of these experiments. **C** The whole-cell lysate was collected for mitotic regulatory proteins, cdk1/cdc2, cyclin B1, PLK, MPM-2, and aurora A, detection by western blotting. The data were repeated for at least three independent experiments. ^∗^*p* < 0.05, **p < 0.01 compared with G_2_/M control (C, untreated) group in MOLT-4 cells; ^##^*p* < 0.01 compared with G_2_/M control (C, untreated) group in HL-60 cells.

We evaluated cell cycle distribution to investigate the influence on cancer cell growth after MPT0G449 treatment. Representative histograms of cell cycle distribution showed that the G_2_/M population increased in a time-dependent manner (Fig. 3A, B). As shown in Fig. 3B, acute leukemia cells accumulated in the G_2_/M phase for 6 to 12 h under MPT0G449 treatment. We also evaluated G_2_/M transition protein expression using western blot assay. Cdk1/cdc2 (Try15) is an inhibitory protein that cannot interact with cyclin B1 to promote cell progression from G_2_ to M phase^40^. The results revealed that MPT0G449 enhanced cdk1/cdc2 (Try15) protein expression and decreased cyclin B1, MPM-2, aurora A, and PLK (T210) expression after 12 h of treatment (Fig. 3C). These results suggest that MPT0G449 induced leukemic cell arrest at the G_2_ phase.

### MPT0G449 significantly induces acute leukemia cell apoptosis through a caspase-mediated pathway

**Fig. 4:**
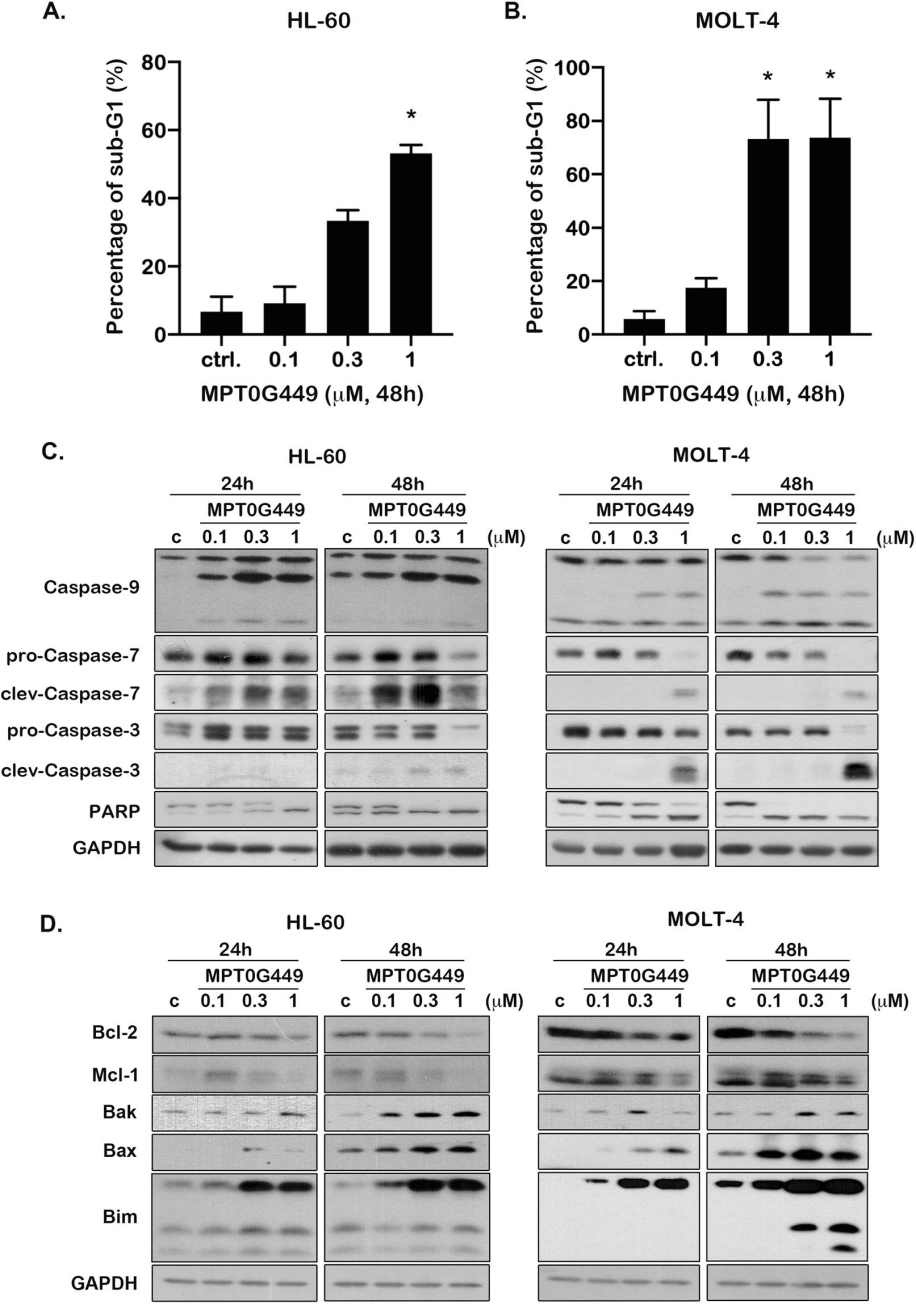
MPT0G449 induces cell apoptosis and activates apoptotic protein expression in HL-60 and MOLT-4 cells. HL-60 **A** and MOLT-4 **B** cells were treated with 0.1, 0.3, and 1 μM MPT0G449 for 48 hours, and the sub-G1 phase was detected by flow cytometry. The results represent the mean ± SD of three independent experiments at ^∗^*p* < 0.05 compared with the control (c, untreated) group. (**C**, **D**) HL-60 and MOLT-4 cells were treated with various concentrations of MPT0G449 (0.1, 0.3, 1 μM) for 24 and 48 h. Cells were then harvested for detection of caspase-9, caspase-7, caspase-3, and PARP activation **C**. After 24 and 48 h MPT0G449 treatment, the level of Bcl-2 related signaling (Bcl-2, Mcl-1, Bak, Bax, and Bim) were then determined **D**. The whole-cell lysates were subjected to western blotting, and the data were repeated at least three independent experiments.

To understand whether apoptosis of leukemic cells was induced after cell cycle arrest, we analyzed the proportion of cells accumulated in the sub-G1 phase. The results showed that MPT0G449 significantly induced cell death in a concentration-dependent manner (Fig. 4A and Supplementary Fig. 2). Cell apoptosis is mainly induced through extrinsic (death receptor) and intrinsic (mitochondrial) pathways. Both pathways lead to activation of the executioner caspases, caspase-3, and caspase-7, leading to programmed cell death^41^. To verify the role of caspase-mediated cell apoptosis, we treated HL-60 and MOLT-4 cells with various concentrations of MPT0G449 at different time courses. The results showed that the intrinsic apoptotic pathway was induced by the activation of caspase-3, 7, and 9 after MPT0G449 treatment. Poly (adenosine diphosphate-ribose) polymerase (PARP) activation was also induced in a time- and concentration-dependent manner (Fig. 4C).

Mitochondria play an important role in cell apoptosis through the participation of Bcl-2 family members. Bim protein interacts with Bcl-2 to allow Bax and Bak proteins to release cytochrome c from the mitochondria to the cytosol, which in turn drives caspase-signaling activation^42–44^. Our results showed that the pro-apoptotic Bcl-2 family proteins Bax, Bak, and Bim were upregulated and the antiapoptotic proteins Bcl-2 and Mcl-1 were downregulated by MPT0G449 treatment (Fig. 4D). Therefore, these data suggested that MPT0G449 induced cell apoptosis by the stimulation of Bcl-2 signaling and a caspase-dependent mechanism.

### MPT0G449 decreases oncogenic signaling in acute leukemia cells

**Fig. 5:**
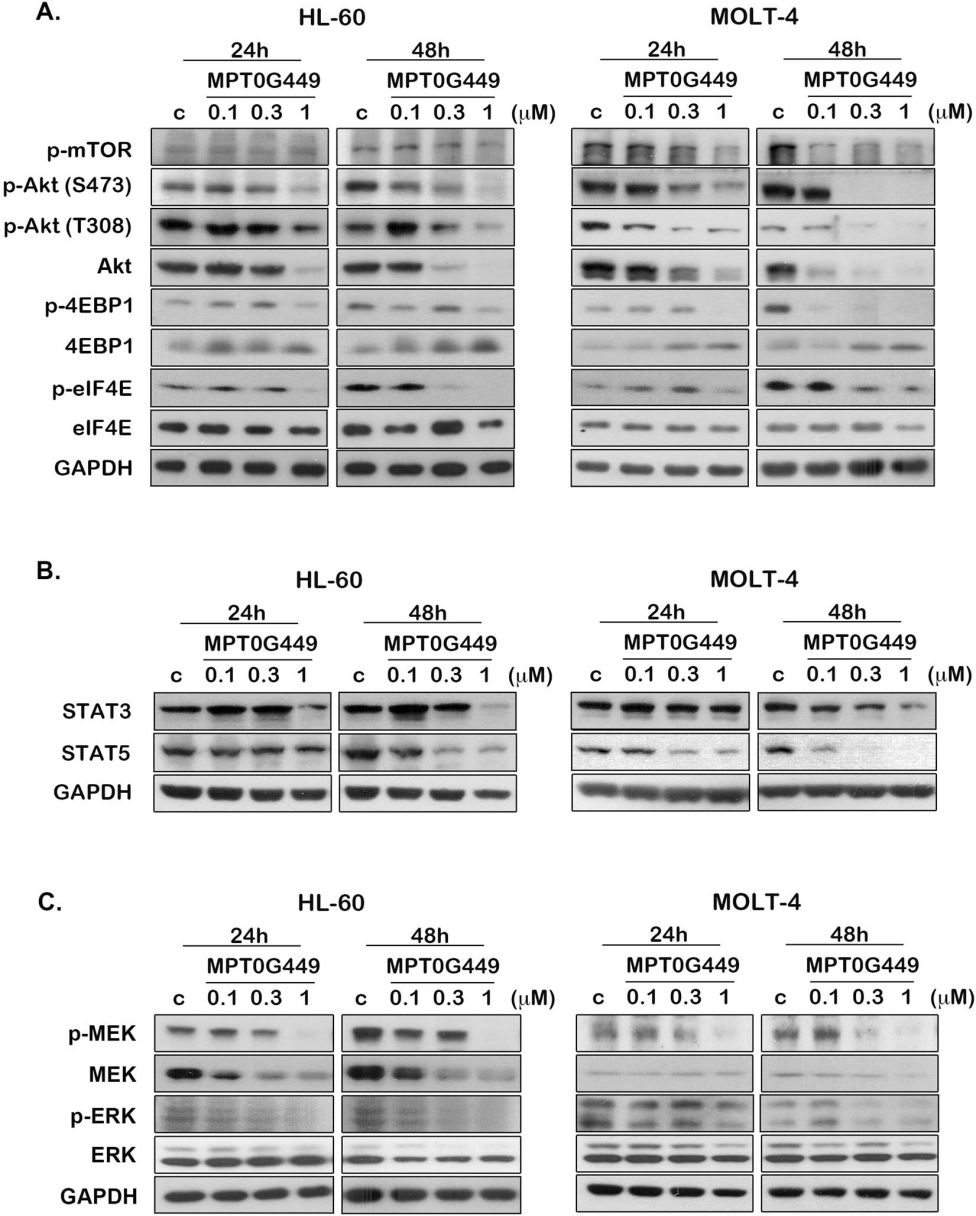
The inhibitory effect of MPT0G449 on AKT/mTOR, STAT, and MEK/ERK signalings. HL-60 and MOLT-4 cells were incubated with MPT0G449 (0.1, 0.3, 1 μM) for 24 or 48 h, then determined **A** AKT/mTOR signaling proteins, p-mTOR, p-Akt (S473), p-Akt (T308), Akt, p-4EBP1, 4EBP1, p-eIF4E and eIF4E, expression. **B** The level of STAT signalings, STAT3 and STAT5, and **C** MEK cascade signalings, p-MEK, MEK, p-ERK, and ERK were determined after MPT0G449 treatment. The whole-cell lysates were examined by western blotting, and these results were repeated at least three independent experiments.

Gene set enrichment analysis (GSEA) analysis revealed that the oncogenic pathways, PI3K/AKT/mTOR and STAT pathway, were highly enriched in AML and ALL gene expression profiles of patients (Supplementary Fig. 3). According to the literature, the PI3K/AKT and JAK/STAT pathways are constitutively activated, which is associated with receptor tyrosine kinase (RTK) mutation in leukemias^45,46^. We, therefore, tested the effect of MPT0G449 on PI3K/AKT/mTOR and STAT signal transduction. P-mTOR, p-AKT (Ser473, Thr308), AKT, and p-4EBP1 protein were markedly decreased in a concentration-dependent manner at both 24 and 48 h after MPT0G449 treatment (Fig. 5A). Additionally, our results also showed that MPT0G449 evidently suppressed STAT3 and STAT5 protein expression (Fig. 5B). Moreover, previous reports have indicated that approximately 30% of adult acute leukemia patients exhibit internal tandem duplications (ITDs) in FLT3 RTK, which results in constitutive activation of the PI3K/AKT, MEK/ERK, and STAT pathways^47,48^, and the MEK/ERK cascade may be induced by chemotherapeutic agents during leukemia therapy, which may contribute to drug resistance^49^. Therefore, we determined that MEK cascade signals, phospho-MEK, MEK, phospho-ERK, and ERK were downregulated after MPT0G449 treatment (Fig. 5C). Together, the dual effect inhibitor MPT0G449 markedly suppresses oncogenic signaling in acute leukemia cells.

### MPT0G449 contributes to tumor growth inhibition in mouse xenograft models

**Fig. 6:**
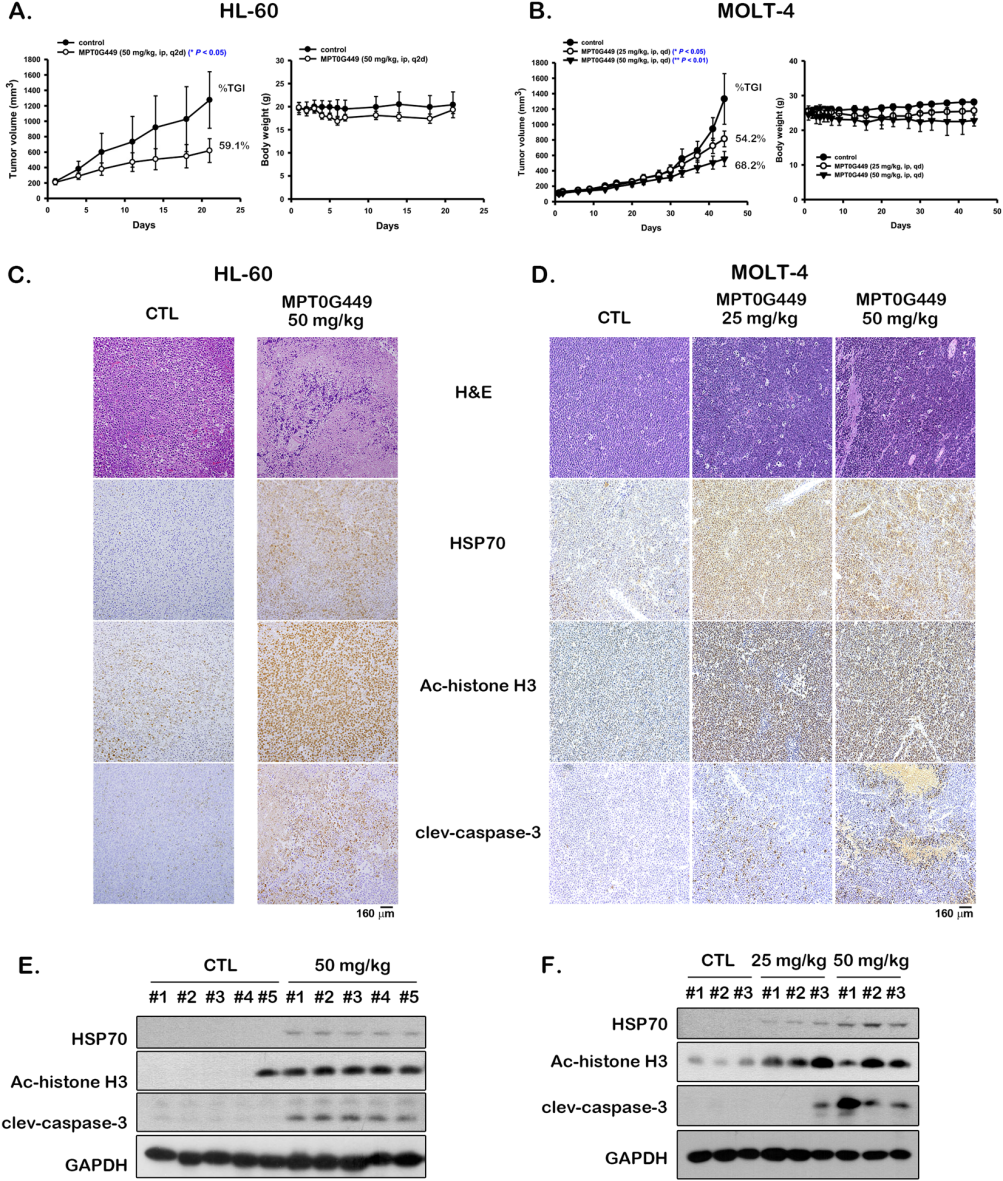
MPT0G449 exhibited anti-tumor activity in HL-60 and MOLT-4 xenograft models. Severe combined immunodeficiency (SCID) mice were subcutaneously injected with 1 × 10^7^ HL-60 or MOLT-4 cells. After tumor size reached approximately 200 mm^3^, mice received vehicle or MPT0G449 (25 or 50 mg/kg by intraperitoneal (i.p.) injection) in HL-60 (**A**) or MOLT-4 (**B**) models. Tumor size was measured twice per week and body weights were measured daily during the first week and then twice per week. Tumor growth inhibition (TGI) was represented as mean ± S.E. *p < 0.05 and **p < 0.01 as compared with the control group. Tumors were harvested at terminal sacrifice and biomarker proteins were subjected to IHC stain and western blotting. Expression levels of biomarkers of MPT0G449 in HL-60 (**C**, **E**) and MOLT-4 (**D**, **F**) xenografts.

To validate the antitumor activity of MPT0G449 in vivo, severe combined immunodeficiency (SCID) mice were subcutaneously injected with HL-60 or MOLT-4 cells. When the tumor reached 200 mm^3^, the mice received MPT0G449 (25 or 50 mg/kg) by intraperitoneal (IP) injection. This experiment was discontinued when the tumor volume reached approximately 1200 mm^3^. In vivo studies showed that MPT0G449 significantly inhibited tumor growth at 50 mg/kg and without body weight loss (Fig. 6A, B). Immunohistochemistry (IHC) staining and western blotting confirmed the antitumor effect of MPT0G449 through HDAC and HSP90 inhibition by the observation of acetyl-histone H3 and HSP70 protein induction (Fig. 6C–F). In addition, MPT0G449 caused apoptosis (cleaved caspase 3 induction) in tumor cells in vivo (Fig. 6C–F). The xenograft experiment indicated that MPT0G449 suppressed tumor growth by HDAC and HSP90 inhibition, suggesting that a dual HDAC and HSP90 inhibitor could be a potent strategy for anticancer treatment.

## Discussion

Acute leukemia is an aggressive hematologic tumor resulting from the rapid differentiation and proliferation of immature blood cells. Despite the high cure rate of pediatric patients using chemotherapy, the treatment of adults has resulted in the survival of ~40%, and the outcome of acute leukemia patients with primary resistant or relapsed leukemia remains poor^50–52^. Therefore, the development of alternative therapeutic strategies against acute leukemia is urgently needed.

Combination treatments are common therapeutic regimens that use two or more drugs to exert synergistic anticancer effects by reducing the development of drug resistance^53,54^. Meanwhile, drug–drug interactions and unexpected side effects might diminish the effectiveness of treatment. Panobinostat, a pan-HDAC inhibitor, combined with dexamethasone and bortezomib, has been approved for the treatment of multiple myeloma. Dexamethasone has been shown to influence the disposition of panobinostat due to dexamethasone as a hepatic enzyme (cytochrome p450 3A4) inducer and panobinostat, which is extensively metabolized by liver enzymes, enhances pharmacokinetic clearance^55,56^. Currently, to promote therapeutic efficacy, researchers have proposed dual/multi-targeted inhibitors for anticancer treatment. For instance, QL-X-138 targets both BTK/MNK kinase and effectively induces apoptosis in lymphoma and leukemia cells^57^, and TAK-659 is a dual SYK/FLT-3 inhibitor that has displayed notable antitumor growth activity in clinical trials for FLT3-dependent AML and advanced solid tumor and lymphoma malignancies^58^. Therefore, the development of a drug with dual/multiple targets is required for leukemia, and this method offers a cost-effective strategy that is beneficial for drug discovery over multi-drug combinations.

HDACs and HSP90 are involved in the activation of several oncogenic pathways in cancer maintenance, including the PI3K/AKT/mTOR, STAT, EGFR, and RAF/MEK/ERK pathways, which were also highly enriched in AML and ALL patient profiles (Supplementary Fig. 3C). Although phosphorylated AKT and STAT3 are downregulated under HDAC inhibitor treatment^59–61^, activation of STAT1, STAT3, and STAT5 has been reported in vorinostat-resistant cells^62,63^. Moreover, MEK/ERK signaling is also highly activated in HDAC inhibitor-resistant cells following exposure to chemotherapeutic agents both in solid tumors and hematological cancer^64–66^. Therefore, single-use HDAC inhibitors are not sufficient for cancer or advanced malignancy treatment. Accordingly, HSP90 inhibitors induce proteasome-mediated degradation of client proteins (EGFR, JAK2, AKT, and Her2/ErbB2), which are essential for cell growth and survival signaling activation^67,68^. Parenthetically, extracellular HSP90 has been shown to enhance the maturation of matrix metalloproteinases and increase cancer cell metastasis^69^. Recent studies have revealed that HDAC inhibitor-resistant cells exhibit a lack of HDAC6 expression and induce localization of hyperacetylated HSP90 on the cell surface; moreover, these cells show sensitivity to HSP90 inhibitors^70^. Many studies have reported that co-treatment with HDAC and HSP90 inhibitors exhibited synergistic anticancer efficacy in different cancer types^26–29^. In our study, MPT0G449 exhibited strong anticancer activity in leukemia (Table 1 and Fig. 6) and showed an extensive inhibitory effect on both phosphorylated and total AKT/mTOR, STAT3/5, and MEK/ERK protein expression (Fig. 5).

MPT0G449 contributed to cell cycle arrest at the G_2_ phase and further accumulated cells in the sub-G1 phase under treatment. Recent studies have indicated that HDAC inhibitors induce cell cycle arrest at the G_0_/G_1_, G_1_/S, or G_2_/M phase depending on the HDAC inhibitors and cell line types^71^. Belinostat (PXD-101) and panobinostat (LBH-589) reduced cyclin E1/cyclin A2 levels and showed EZH2 depletion, leading to S phase arrest in HL-60 cells^72,73^. Vorinostat (SAHA) induced cell cycle arrest at G_2_/M by decreasing cyclin D1 and cyclin B expression, which was consistent with our studies (Fig. 3). This is because the HDAC inhibitory group of MPT0G449 was designed based on vorinostat. Another notable finding is that MPT0G449 not only decreased cyclin B1 expression but also reduced aurora A protein levels (Fig. 3C). Aurora A, a mitotic serine/threonine kinase, is implicated in the mitosis and meiosis of cell proliferation. Additionally, aurora A has been shown to be a client protein of HSP90, which facilitates aurora A degradation after HSP90 inhibitor treatment^74^. Accordingly, we suggest that MPT0G449 inhibits HSP90 activity to decrease aurora A expression. Based on this evidence, MPT0G449 targets both HDAC and HSP90 to induce cell cycle arrest at the G_2_ phase, which was expected.

In conclusion, we evaluated a dual effect inhibitor, MPT0G449, which targets HDAC and HSP90 enzyme inhibition, and remarkably induces cell apoptosis in acute leukemia cells. MPT0G449 suppresses oncogenic signaling (AKT/mTOR, STAT3/5, and MEK/ERK) expression, which is highly enriched in AML and ALL patient gene profiles. Moreover, MPT0G449 exhibited notable antitumor growth activity in HL-60 and MOLT-4 xenograft models. This is a proof-of-concept pre-clinical study to elucidate the anticancer activity of a dual HDAC and HSP90 inhibitor and suggests that MPT0G449 represents a novel therapeutic approach for acute leukemia treatment.

## Materials and methods

### Reagents and antibodies

A series of novel dual HDAC and HSP90 inhibitors were synthesized by Dr. Jing-Ping Liou (School of Pharmacy, College of Pharmacy, Taipei Medical University, Taiwan). 3-(4,5-dimethylthiazol-2-yl)-2,5-diphenyltetrazolium bromide (MTT), and propidium iodide (PI) were purchased from Sigma Chemical Co. (St. Louis, MO, USA). Primary antibodies MPM2 (pSer/pThr) (05-368) and GAPDH (MAB374) were purchased from Millipore (Bedford, MA, USA). Acetyl-histone H3(CS-9649S), histone H3 (CS-9715), acetyl-α-tubulin (CS-5335S), α-tubulin (CS-2125S), HDAC1 (CS-5356), HDAC2 (CS-5113), HDAC6 (CS-7558), HDAC8 (CS-66042S), HSP90 (CS-4874), HSP70 (CS-4876), aurora A (CS-3092S), cleaved-caspase 3 (CS-9661), PARP (CS-9452S), caspase 9 (CS-9502S), Bcl-2 (CS-2876S), Bak (CS-3814S), Bax (CS-2772S), Bim (CS-2819S), p-mTOR (CS-2974), p-Akt (Ser473) (CS-4060S), p-Akt (Thr308) (CS-13038S), Akt (CS-9272S), p-4EBP1 (CS-9455), 4EBP1 (CS-9644), p-eIF4E (CS-9741), eIF4E (CS-2498), STAT5 (CS-4807S), p-MEK (CS-9154), MEK (CS-9122), p-ERK (CS-9101) and ERK (CS-9102) were purchased from Cell Signaling Technologies (Beverly, MA, USA). Cyclin B1 (554176), Cdk1/cdc 2(Tyr15) (612306), caspase 7 (556541) and STAT3 (610190), were purchased from BD (Biosciences, USA). Mcl-1 (SC-819) was purchased from Santa Cruz (Dallas, USA). The labeled secondary antibodies goat anti-rabbit IgG-HRP (111-035-003) and goat anti-mouse IgG-HRP (115-035-003) were purchased from Jackson ImmunoResearch (Jackson ImmunoResearch, WG, USA).

### Cell lines

Human acute myeloid leukemia cell line, HL-60, human acute lymphoblastic leukemia cell line, MOLT-4, and retinal pigmented epithelium cell line, ARPE-19, were purchased from American Type Culture Collection (ATCC, Manassas, VA, United States). HL-60 cells were maintained in IMDM with 20% (v/v) inactive fetal bovine serum, and MOLT-4 cells were maintained in RPMI-1640 with 10% (v/v) inactive fetal bovine serum. ARPE-19 cells were cultured in DMEM/F12 with 10% (v/v) fetal bovine serum. Human chronic myelogenous leukemia cell line, K562, and umbilical vein/vascular endothelium cell line, HUVEC, were purchased from Bioresource Collection and Research Center (BCRC, Hsinchu, Taiwan). K562 cells were cultured in IMDM with 10% fetal bovine serum. HUVEC cells were cultured in endothelial cell growth supplement (ECGS) with 10% (v/v) fetal bovine serum. All culture mediums contained penicillin (100 units/mL) and streptomycin (100 μg/mL, Biological Industries Ltd., Kibbutz Beit HaEmek, Israel). Human peripheral blood mononuclear cells, PBMCs, were purchased from STEMCELL Technologies (Vancouver, Canada) and cultured in DMEM with 10% fetal bovine serum. All cells were incubated in an incubator in the presence of 5% CO_2_ at 37 °C. All cell lines have been authenticated using STR profiling and tested for mycoplasma contamination by Genelabs life science (Genelabs Life Science corp., Taipei, Taiwan).

### MTT assay

MOLT-4 and HL-60 cells were seeded in 24-well plates at a density of 4 × 10^5^ cells/well with 1 mL culture medium then treated with different concentrations of MPT0G449 for 48 h. Cell viability was determined by treating the cells with MTT (0.5 mg/mL in PBS) for 1 h at 37 ˚C. The crystal formazan dyes were then dissolved in 1 mL sodium acetate buffer^75^. The absorbance was spectrophotometrically analyzed at 550 nm by an ELISA reader (Molecular Devices, Sunnyvale, CA, USA).

### HDAC activity assay

HDAC isoforms’ inhibition activity was served by RBC (Reaction Biology Corp., Malvern, USA). We provided the MPT0G449 compound to Uni-Onward Corp. (Taipei, Taiwan), then entrusted RBC for isoform activity detection.

### Molecular docking analysis

The molecular docking analysis was performed using the docking software LeadIT^76^. The crystal structures of HSP90α (PDB ID: 2CCU) and HDAC6 (PDB ID: 5EDU) were downloaded from the Protein Data Bank^77^. The structures were then prepared by LeadIT and water molecules were removed. The protonation form of MPT0G499 was generated in an aqueous solution. The binding sites were defined as 15 Å from the co-crystallized ligands. The docking strategy was based on an enthalpy and entropy approach. The maximum number of solutions for both the iteration and fragmentation were set at 500. All other parameters used the default setting.

### Flow cytometry

The cell cycle as evaluated by flow cytometry. MOLT-4 and HL-60 cells (2 × 10^6^ cells/well) were seeded in 6-well plates in a 2 mL culture medium and treated with a gradient concentration of MPT0G449 for the indicated periods. After drug treatment, cells were collected, washed with cold PBS, and fixed with 70% (v/v) ice-cold ethanol at −20 ˚C for 30 min. The fixed cells were centrifuged to remove the ethanol, resuspended in 0.1 mL DNA extraction buffer (0.2 M Na_2_HPO_4_-0.1 M citric buffer, pH 7.8) for 20 min. The cells were centrifuged and stained with 0.5 mL PI staining buffer (80 μL/mL PI, 100 μL/mL RNase A and 1% Triton X-100 in PBS) for 30 minutes. Cell cycle distribution was analyzed by BD FACScan Flow Cytometer and CellQuest software and BD Accuri^TM^ Flow cytometer and software (Becton Dickinson, Mountain View, CA, USA).

### Western blot analysis

Cells were incubated with various concentrations of MPT0G449, harvested, washed with PBS, lysed in lysis buffer (50 mM Tris pH 7.4, 150 mM NaCl, 1% Triton X-100, 1 mM EDTA, 1 mM EGTA, 1 mM PMSF, 10 μg/mL aprotinin, 10 μg/mL leupeptin, 1 mM sodium orthovanadate, and 1 mM NaF), and then centrifuged for 30 min at 14,000 rpm at 4 ˚C. The harvested total protein was quantified by BCA Protein Assay Kit (Thermo Fisher Scientific, Rockford, IL, USA). Whole-cell extracts were mixed with 5 × sample buffer (312.5 mM Tris pH 6.8, 10% SDS, 50% glycerol, 0.05% bromophenol blue, and 10% 2-mercaptoethanol) at 95 ˚C for 10 min. An equal amount of total protein samples was separated by SDS-PAGE and subsequently transferred onto PVDF membranes. The membranes were blocked with 5% non-fat milk in PBS for 1 h at room temperature and incubated with primary antibodies in PBST buffer (0.1% Tween 20 in PBS) at 4 ˚C overnight. The membranes were washed with PBST, followed by incubation with the corresponding HRP-conjugated secondary antibodies diluted in 0.5% non-fat milk in PBST for 1 h at room temperature. Bound antibodies were measured using an enhanced chemiluminescence detection kit (Amersham, Buckinghamshire, UK).

### In vivo xenograft model

To evaluate the antitumor activity of MPT0G449, 4-week-old male SCID mice were subcutaneously injected with 1 × 10^7^ leukemic cells (HL-60 or MOLT-4). When the tumor sizes reached 200 mm^3^, mice were randomly distributed into three treatment groups (5 mice in each group). MPT0G449 was applied with indicated dosage (25 or 50 mg/kg) by intraperitoneal injection (i.p.), once per every other day (q2d) for HL-60 and once daily (qd) for MOLT-4 models. During the experiment, the tumor size and body weight were measured twice each week, and the tumor volume (mm^3^) was calculated as *LW*^*2*^ / 2 (*L* is tumor length, and *W* is tumor width). Mice were ethically sacrificed when the tumor volume reached 1200 mm^3^. Animal experiments were performed in accordance with relevant guidelines and regulations followed by ethical standards, and protocols has been reviewed and approved by the Animal Use and Management Committee of Taipei Medical University. (IACUC number: LAC-2015-0240).

### Immunohistochemistry (IHC) staining

Tumor tissues were removed from SCID mice, then we provided acetyl-histone H3, HSP70, and cleaved-caspase 3 antibody to Rapid Science Co. Ltd. (Taipei, Taiwan) for immunohistochemistry staining service. Histopathological evaluation was assessed by TissueFAXS (TissueGnostics, Vienna, Austria).

### Systematic analysis

The gene expression profiles of AML (GSE110087), T-ALL patients (GSE87865) and healthy human hematopoietic stem cells (HSC, GSE32719) were searched from NCBI Gene Expression Omnibus (GEO) website (https://www.ncbi.nlm.nih.gov/geo/). The pathways and oncogenic signatures were enriched by Gene Set Enrichment Analysis (GSEA) (https://www.gsea-msigdb.org/gsea/index.jsp). The compound prediction was analyzed on LINCS L1000 Connectivity Map (CMAP) library (https://clue.io).

### Data analysis and statistics

As indicated in the figure legends, the sample size of the experiments depended on the assay type. There were no blind experiments for the investigators both in cells and mice experiments. All experiments were done three times independently with the data presented as mean ± SD and GraphPad Prism Software version 8.0. Kruskal–Wallis test were performed to compare difference of the medians between all condition groups. The student’s *t* test was used for the comparison of two groups. The animal experiments were determined by the Mann–Whitney test. Parameters with *p-value* < 0.05 are considered statistically significant.

## Supplementary information

Supplementary Figure and Table Legends

Supplementary table 1

Supplementary table 2

Supplementary figure 1

Supplementary figure 2

Supplementary figure 3
