# Supplementary material for: A novel dual HDAC and HSP90 inhibitor, MPT0G449, downregulates oncogenic pathways in human acute leukemia in vitro and in vivo
Source: Oncogenesis. 2021 May 13;10(5):39. doi: 10.1038/s41389-021-00331-0 (PMC8119482; doi:10.1038/s41389-021-00331-0)
Supplement: Supplementary file 1 — Supplementary Figure and Table Legends [file 41389_2021_331_MOESM1_ESM.docx]

**Supplementary Figure and Table Legends**

**Supplementary table 1. The comparison of the current series of compounds with the published compounds in EJMC 2020.**

**Supplementary table 2. The cytotoxic effect of MPT0G449 in human normal and cancer cell lines.**

**Supplementary figure 1. The expression of target proteins and cell viability in HL-60 and MOLT-4 cells.**

(A) The expression of HDAC1, 2, 4, 6, 8, and HSP90 proteins were determined in PBMC, HL-60, MOLT-4 and K562 cells. (B) HL-60 and MOLT-4 cells were incubated with MPT0G449 (1 μM) for 1, 6, 12, 24 hours, and cells were harvested for detection of HDAC1, 2, 4, 6 and 8 protein expressions. The whole cell lysates were examined by western blotting. (C) Cell viability was measured in acute leukemia cell lines, HL-60 and MOLT-4, with 1μM MPT0G449 for 1, 6, 12, 24 and 48 hours treatment. The results represent the mean ± SD of three independent experiments.

**Supplementary figure 2. MPT0G449 induces leukemia cell apoptosis.**

(A) The histograms of cell cycle distribution were detected by flow cytometry. HL-60 and MOLT-4 cells were treated with 0.1, 0.3 and 1 μM MPT0G449 for 48 hours.

**Supplementary figure 3. The gene enrichment and compound prediction were analyzed by GSEA and CMAP tools in AML and ALL patient profiles.**

The gene expression profiles were downloaded from NCBI Gene Expression Omnibus (GEO). (A) There are 3185 genes which highly expressed in both AML and ALL patients versus with normal human hematopoietic stem cells. These genes were upon 1.5-fold change expression in patient samples for further investigation. (B) HSP90, HDAC, EGFR, mTOR, AKT, JAK, MEK and RAF were predicted as potential therapeutic targets for AML and ALL treatment by LINCS L1000 Connectivity Map (CMAP) library analysis. (C) These 3185 genes were determined the pathway enrichment by Gene Set Enrichment Analysis (GSEA).
