# Supplementary material for: A novel dual HDAC and HSP90 inhibitor, MPT0G449, downregulates oncogenic pathways in human acute leukemia in vitro and in vivo
Source: Oncogenesis. 2021 May 13;10(5):39. doi: 10.1038/s41389-021-00331-0 (PMC8119482; doi:10.1038/s41389-021-00331-0)
Supplement: Supplementary file 2 — Supplementary table 1 [file 41389_2021_331_MOESM2_ESM.docx]

| **Supplementary table 1.** The comparison of the current series of compounds with the published compounds in EJMC 2020. | |
| --- | --- |
| **Compound** | ***Eur J Med Chem. 2020 Jan. 1*(30)**  **Compound number** |
| **MPT0G313** | **17** |
| **MPT0G314** | **20** |
| **MPT0G315** | **22** |
| **MPT0G316** | **18** |
| **MPT0G317** | **21** |
| **MPT0G446** | **19** |
| **MPT0G447** | **24** |
| **MPT0G448** | **25** |
| **MPT0G449** | **26** |
