# Supplementary material for: A novel dual HDAC and HSP90 inhibitor, MPT0G449, downregulates oncogenic pathways in human acute leukemia in vitro and in vivo
Source: Oncogenesis. 2021 May 13;10(5):39. doi: 10.1038/s41389-021-00331-0 (PMC8119482; doi:10.1038/s41389-021-00331-0)
Supplement: Supplementary file 3 — Supplementary table 2 [file 41389_2021_331_MOESM3_ESM.docx]

| **Supplementary table 2. The cytotoxic effect of MPT0G449 in human normal and cancer cell lines.** | | | | | | |
| --- | --- | --- | --- | --- | --- | --- |
| **Comp.** | **Cell lines (IC_50_ ± SD, μM)** | | | | | |
|  | **PBMC** | **ARPE19** | **HUVEC** | **HL60** | **MOLT4** | **K562** |
| **MPT0G449** | **>10** | **>10** | **>10** | **0.19 ± 0.04** | **0.11 ± 0.03** | **0.3 ± 0.17** |
