# Supplementary figures and images for: A novel dual HDAC and HSP90 inhibitor, MPT0G449, downregulates oncogenic pathways in human acute leukemia in vitro and in vivo
Source: Oncogenesis. 2021 May 13;10(5):39. doi: 10.1038/s41389-021-00331-0 (PMC8119482; doi:10.1038/s41389-021-00331-0)

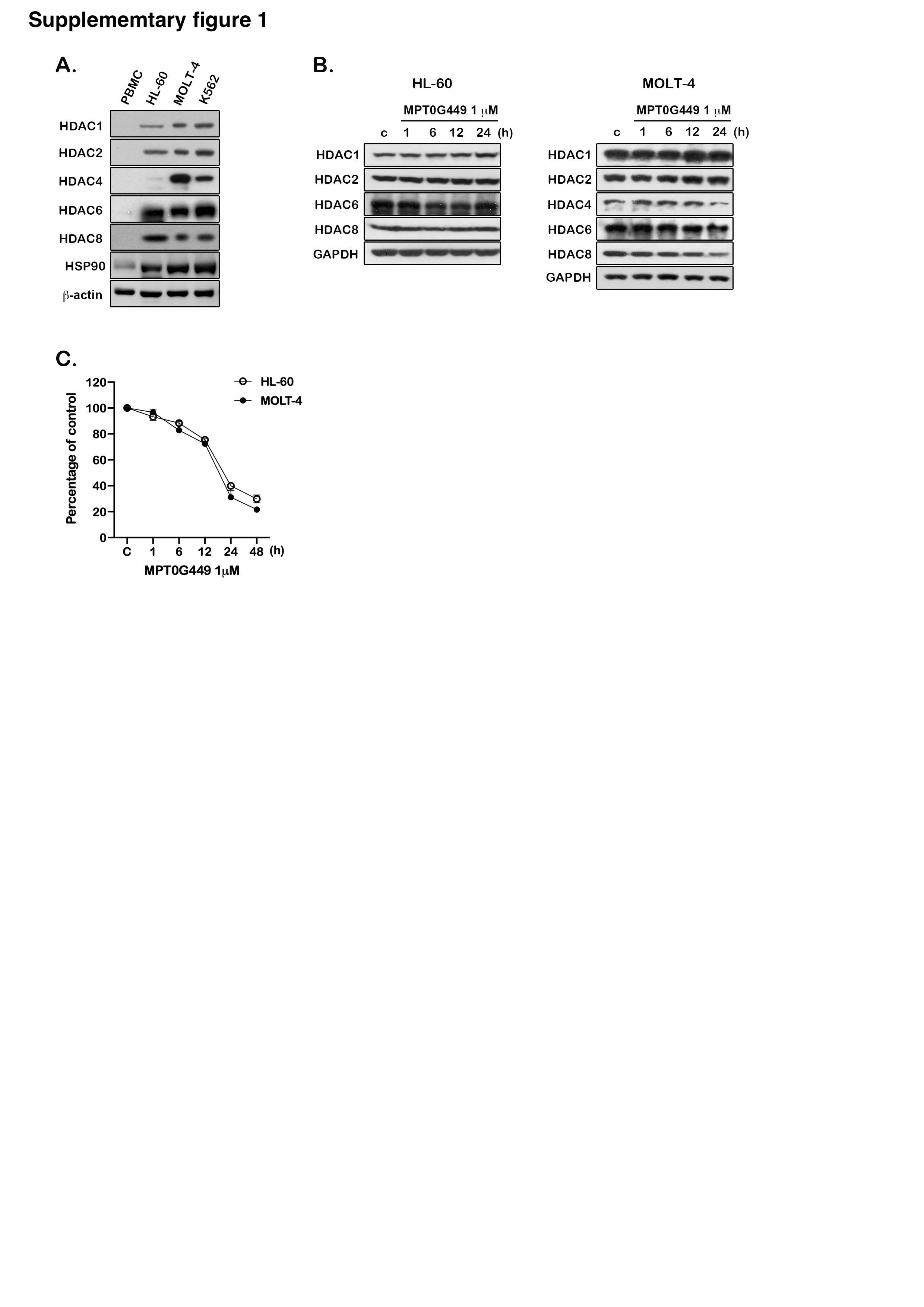

Supplement: Supplementary file 4 — Supplementary figure 1 [file 41389_2021_331_MOESM4_ESM.jpg]

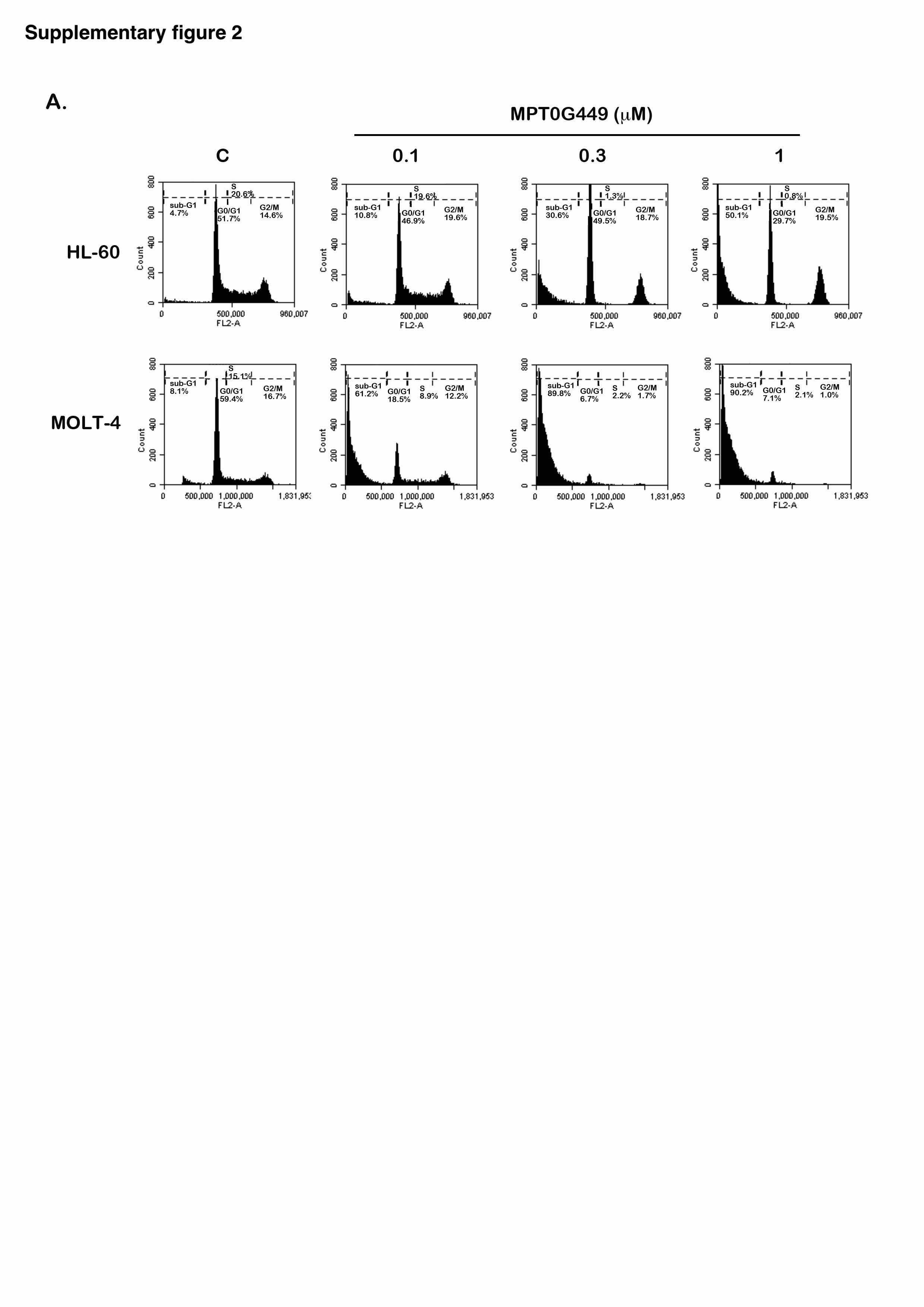

Supplement: Supplementary file 5 — Supplementary figure 2 [file 41389_2021_331_MOESM5_ESM.jpg]

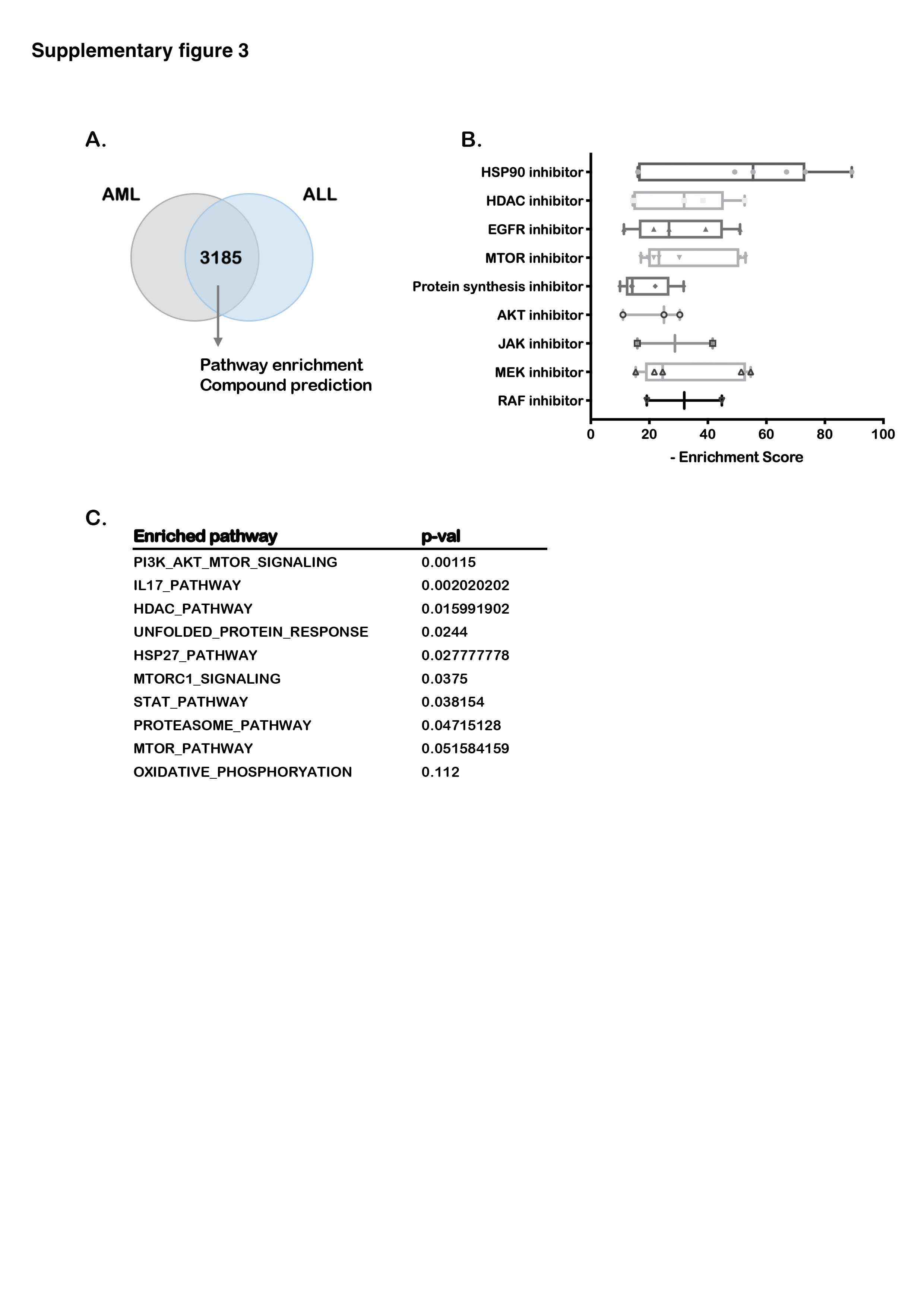

Supplement: Supplementary file 6 — Supplementary figure 3 [file 41389_2021_331_MOESM6_ESM.jpg]
